# Supplementary material for: Structural characterization of HypX responsible for CO biosynthesis in the maturation of NiFe-hydrogenase
Source: Commun Biol. 2019 Oct 18;2:385. doi: 10.1038/s42003-019-0631-z (PMC6802093; doi:10.1038/s42003-019-0631-z)
Supplement: Supplementary file 1 — Supplementary information [file 42003_2019_631_MOESM1_ESM.pdf]

a ATGCGCATCCTGTTTCTGTCGTATCGATTCAATTCATTGTCACAGCGCTTGTATTGCGAACTTACCGAACGCGAACAC
GAAGTTAGCGTGGAATTAGACGTCCATCCTGATCTAACAGTGGAAGCTGCCGAACTGTACAAGCCGGATTTGATTATA
GCTCCGTTTCTTAAACGGAAAATTCCGCAGGAAGTCTGGAAAAAGTACAAAACGCTGATCATTCATCCTGGTCCGCCG
GGAGATCGTGGTCCGAATGCGCTCGATTGGGCGATCATGAAAGGGGAACGAATTTGGGGCGTCACTCTGCTGGAAGCA
AGTGAAGAATATGATGCAGGTGATGTTTGGGCGTACCGTACCTTTCCTATGCGCTTTGCGCGTAAAGCCTCAATTTAT
CGGAATGAAGTGACGGAAGGCGTAGTGGAATGTGTGTTGGAAGCCCTTGAAAACTTCGAGCGGGGGGATTTTAAGCCA
ACACCGCAAAAGGAACATTGGTGGAACCCGAAAATGGAGCAGGAGTTACGTCGTGTTGATTGGGAACAGGATGACACC
AAAACAGTACTCCGGAAAGTCTATGCGTCTGACAGTCAACCAGGTGCCAGCAGCAAAGTCCTAGGTAAAGAGGTCCTC
CTGTTTAATGCGTACCCGGAAGAAGAACTGAAAGGCAAACCAGGTGAAGTGTTAGCATTGCGCGATGAAGCCGTTTGC
ATTGGGACGCGTGATGGAGCCGTATGGATCACTCACATGAGGGAGCGTAAAAAAGAGAGCATTAAACTGCCGTCTGCC
CGTGTGTTAGGCGAGTTCCTCAAAGGCGTGAAAGAAGATCCGATTAAGCCTTGGGAAAAAGTGGACTTTAAAACGTAT
CGCGAAATCCTGTATGAGGAGGAAGACGGCATAGGCTTCATTCACTTCAACTTCTATAACGGTGCGATGTCCACCGAG
CAGTGTTATCGTCTTCTGGAAACCATCAAATACGCCAAAAAACGCCCGGTCAAAGCAATTGTGTTACTGGGTTCTGAG
GATTTTTTCTCGAATGGAATGAACCTGAACACCATTGAGAATGCGGAGAGTCCCGCAGACGAATCGTGGCGCAACATC
AACGCTATCGACGATGTGTGCGAAGAGATCCTTAAAACCCCCGACAAATTAACGGTTGCTGGCATGCAAGGCAATGCC
GGTGCAGGCGGTGTTTTCCTCGCACTGACCTGCGATCTGGTTTTTGCGCGCGAAGGTGTAGTTTTGAATCCCCACTAC
AAAAATATCGGCAATCTGTATGGCTCCGAGTTTTGGACCTATACGCTGCCAAAACGCGTAGGGTGGGAAAAGGGCAAA
GAAGTGATGGAAAACCGTATGCCGATTTCCAGCAAAAAAGCGTTTGAAATTGGTCTGATTGACGGCGTTTTCGGAAAA
ACCCCGAAAGAGTTTCGTCAGCGTCTGAAGGAACGCATCAAAAACTTTATTAACAGCAAAGATTTCTACGAGTTTATC
GAAAAGAAGAAAAAGGAGCGTACTAGTGGGGAATGGCTGGAGGAAATCCAGAAGTGTCGCGAACATGAACTGGAAAAA
ATGAAGCTGAACTTTTATGGCTTCGACACCAGCTACCATATTGCTCGCTACTACTTTGTGCGCCGTAAACCCCACTTT
CGTACTCCACCGTATCTGGCGATCCATCGCAGACTGAAATTCTCGCTGTGA

b MRILFLSYRFNSLSQRLYCELTEREHEVSVELDVHPDLTVEAAELYKPDLIIAPFLKRKIPQEVWKKYKTLIIHPGPP
GDRGPNALDWAIMKGERIWGVTLLEASEEYDAGDVWAYRTFPMRFARKASIYRNEVTEGVVECVLEALENFERGDFKP
TPQKEHWWNPKMEQELRRVDWEQDDTKTVLRKVYASDSQPGASSKVLGKEVLLFNAYPEEELKGKPGEVLALRDEAVC
IGTRDGAVWITHMRERKKESIKLPSARVLGEFLKGVKEDPIKPWEKVDFKTYREILYEEEDGIGFIHFNFYNGAMSTE
QCYRLLETIKYAKKRPVKAIVLLGSEDFFSNGMNLNTIENAESPADESWRNINAIDDVCEEILKTPDKLTVAGMQGNA
GAGGVFLALTCDLVFAREGVVLNPHYKNIGNLYGSEFWTYTLPKRVGWEKGKEVMENRMPISSKKAFEIGLIDGVFGK
TPKEFRQRLKERIKNFINSKDFYEFIEKKKKERTSGEWLEEIQKCREHELEKMKLNFYGFDTSYHIARYYFVRRKPHF
RTPPYLAIHRRLKFSL

c

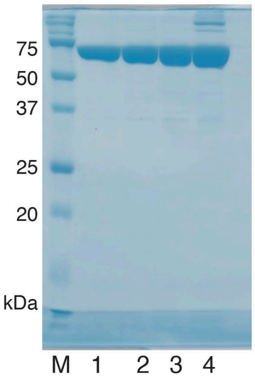

d

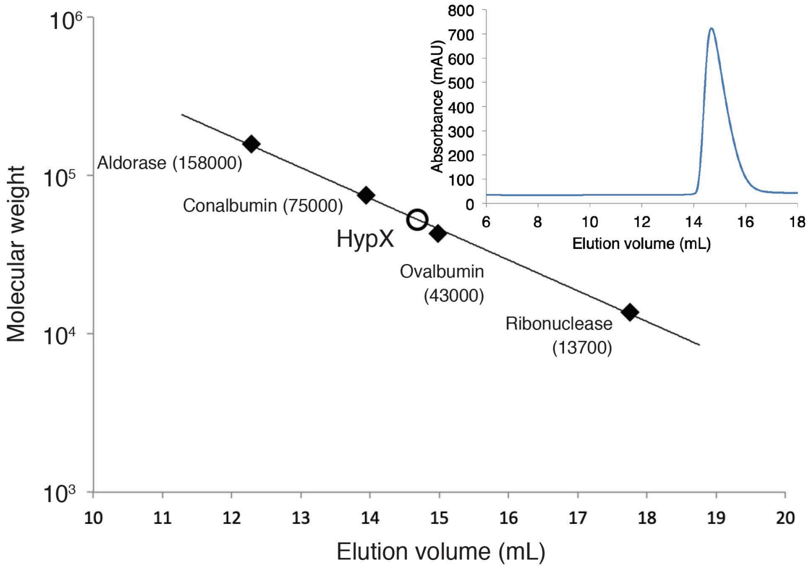

Supplementary Fig. 1. (a) DNA and (b) amino acid sequences of HypX. (c) SDS-PAGE of HypX. M: molecular weight markers, lane 1: wild type HypX, lane 2: R9A-Q15A-R131A-R542A variant, lane 3: Q15A-R131A-S194AQ195A-N306A-R542A variant, lane 4: A392F-I419F variant. (d) Estimation of the mass of HypX. The closed squares show the standard proteins. The vertical axis is in logarithmic scale. (Inset) Elution profile of HypX from a Superdex 200 10/300GL column. HypX (65.5 kDa in monomer) was eluted with an apparent mass of 52.7 kDa, indicating that HypX exists as a monomer in solution.

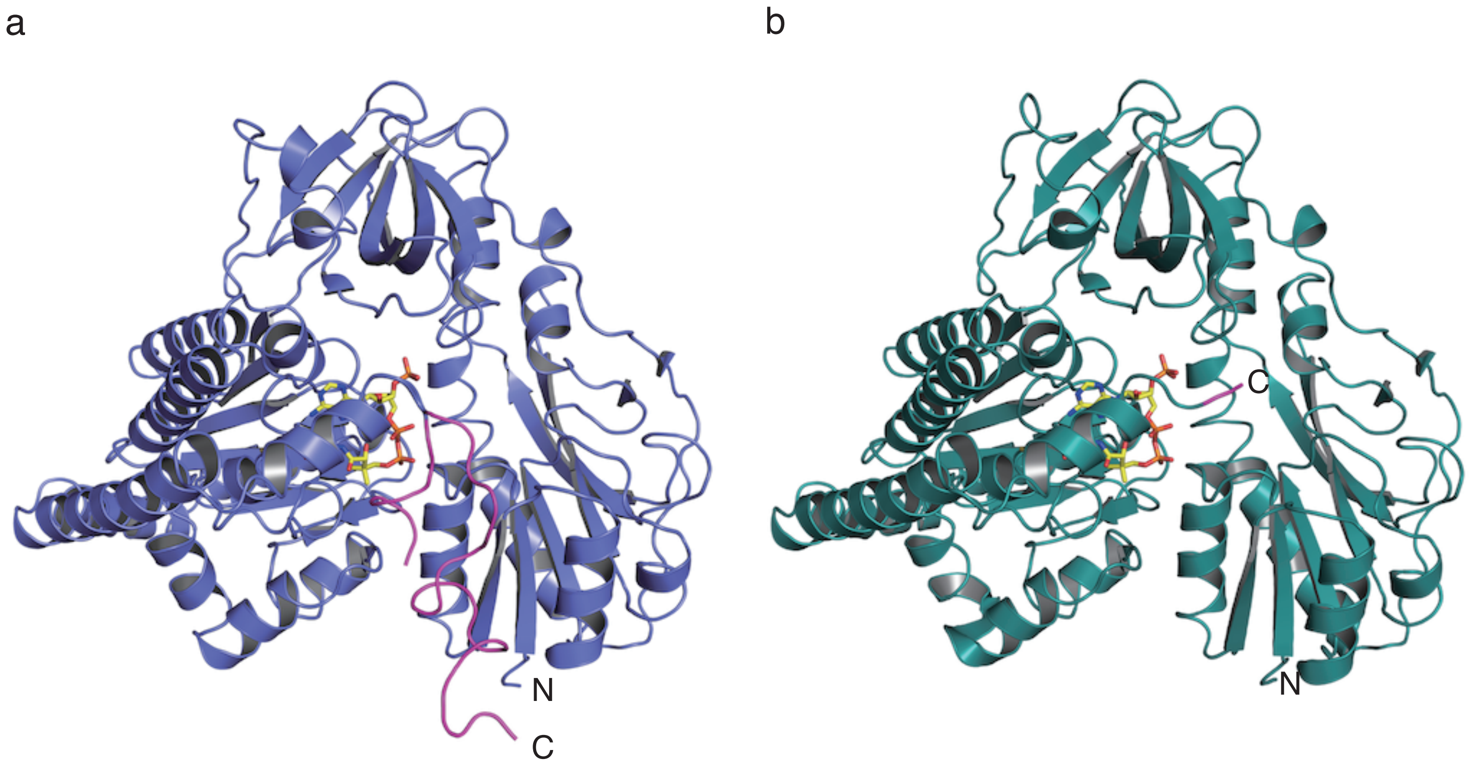

Supplementary Fig. 2. Overall structure of HypX. (a) Chain B of crystal form I (PDB ID: 6J0P) and (b) crystal form II (PDB ID: 6J1E). The r.m.s.d. values were 0.462 Å, 0.414 Å, and 0.596 Å between the chains A and B, the chain A and Form II, and the chain B and Form II, respectively. While clear electron density was observed from the N- to C-terminus in chain A of Form I, the electron density of the C-terminal 18 residues (His545-Leu562) was not observed in Form II. Overall structures of Form I (chains A and B) and Form II were same.

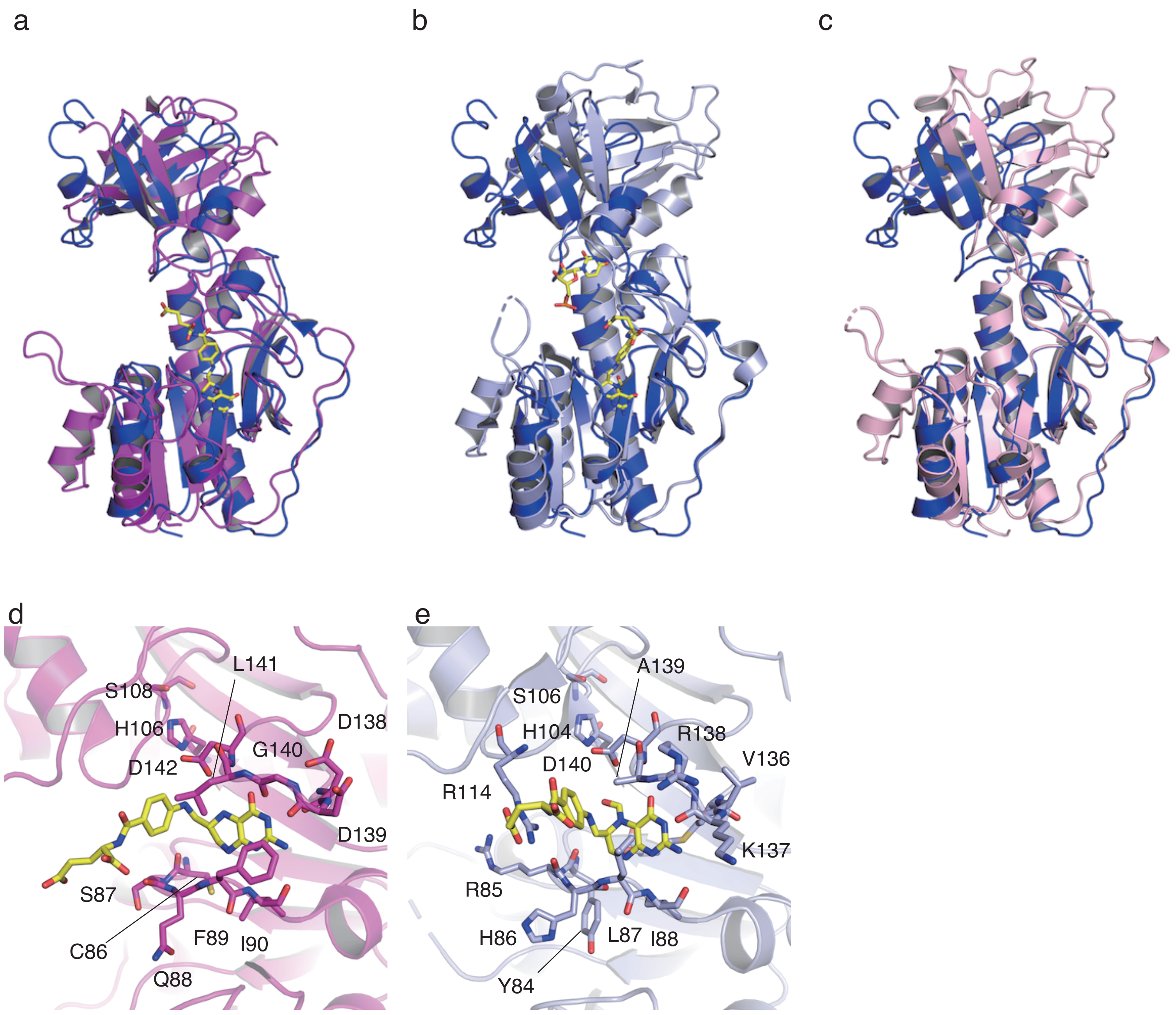

Supplementary Fig. 3. Superposition of the N-terminal domain of HypX (blue) with (a) FDH (purple), (b) ArnA (gray), and (c) FMT (pink). The structures of FDH and ArnA are the THF-bound form. Close-up view of the THF binding region in (d) FDH and (e) ArnA.

a

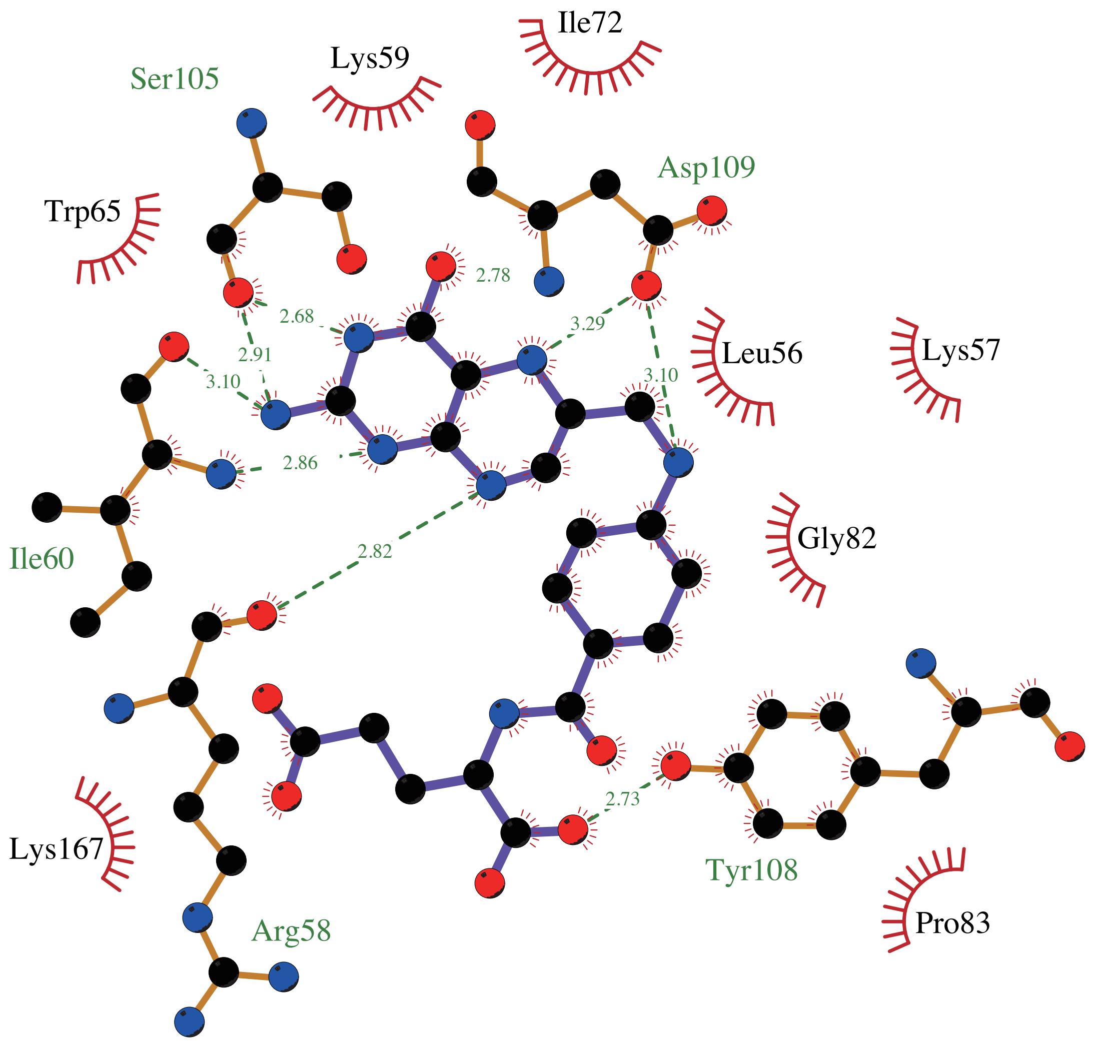

b

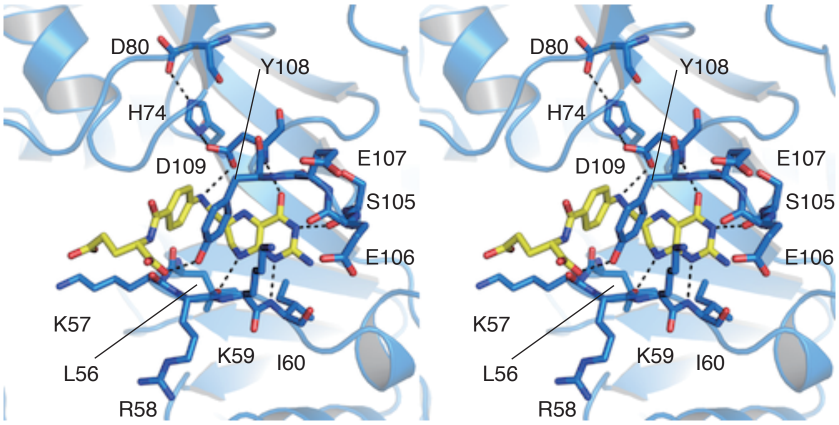

Supplementary Fig. 4. (a) Interaction diagrams for THF and HypX interactions, which were drawn by LigPlot+ (Laskowski, R. A., Swindells, M. B. LigPlot+: multiple ligand-protein interaction diagrams for drug discovery. J. Chem. Inf. Model., 51, 2778-2786 (2011)). (b) Stereo view of the THF binding region in the N-terminal domain of HypX. Hydrogen bonds are shown in dashed lines.

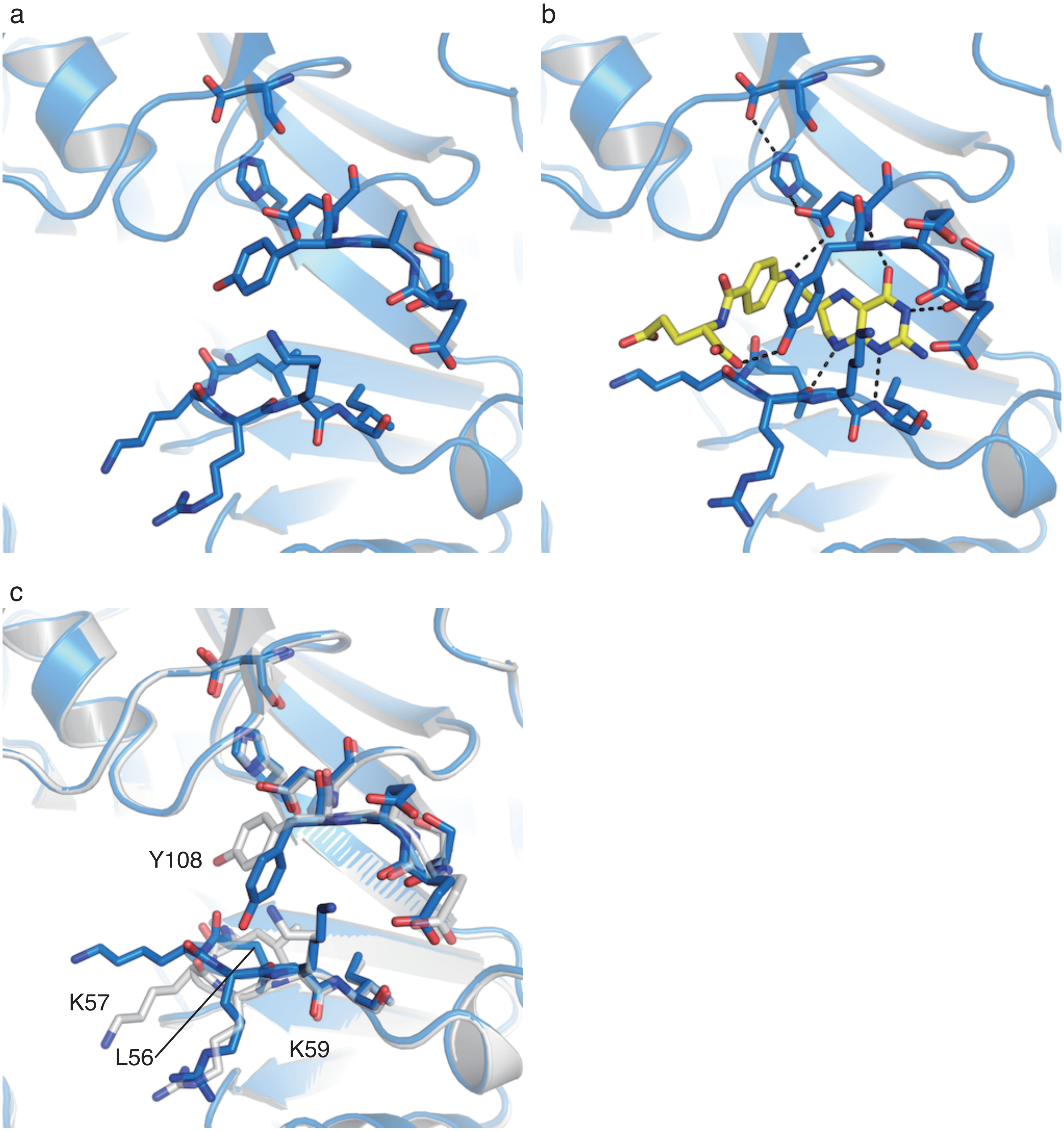

Supplementary Fig. 5. Conformational change of wild type HypX upon THF binding. Close-up view of the THF binding region (a) in the THF-free and (b) in the THF-bound forms. (c) Superposition of (a) and (b). THF is omitted for clarity. The structures of (a) and (b) are shown in blue and light gray, respectively.

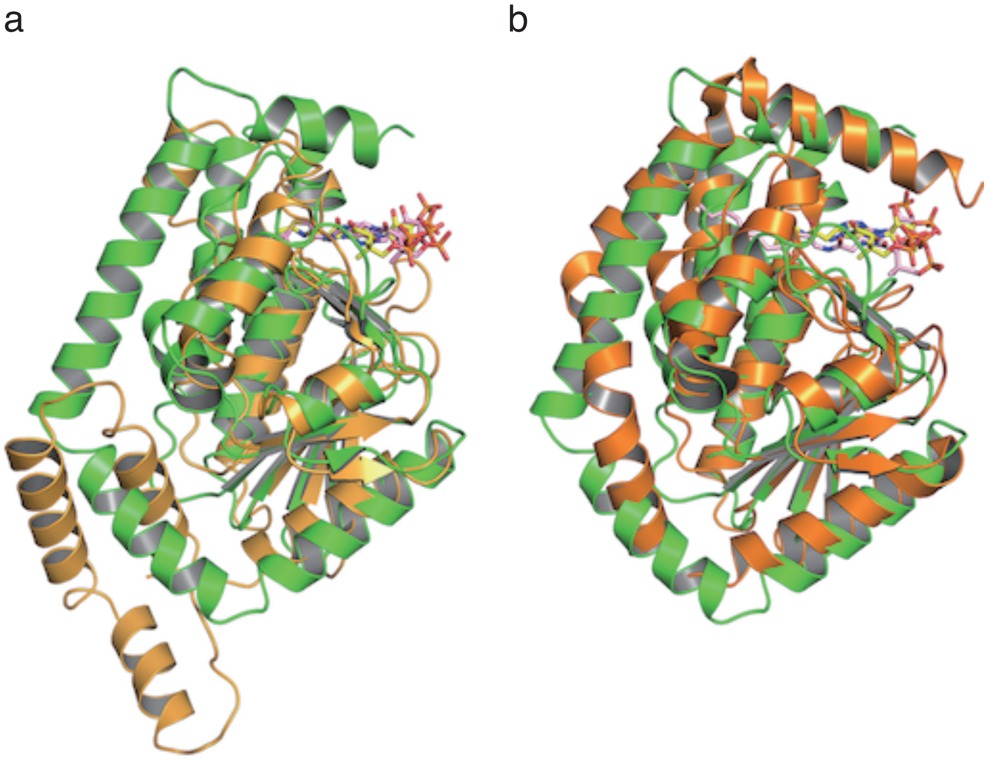

Supplementary Fig. 6. Superposition of the C-terminal domain of HypX (green) with (a) the core domain of ECH (brown, PDB ID: 1dub) and (b) Δ3-Δ2-enoly-CoA isomerase (brown, PDB ID: 1sg4). The r.m.s.d. values compared with the C-terminal domain of HypX are 2.24 Å for the both of ECH and ECI. While HypX and ECI (PDB ID: 1sg4) are monomer, ECH forms a homotrimer in which the C-terminal three helices are domain-swapped (PDB ID: 1dub). Therefore, the core region except for the C-terminal three helices in ECH was used to calculate the r.m.s.d. value between HypX and ECH.

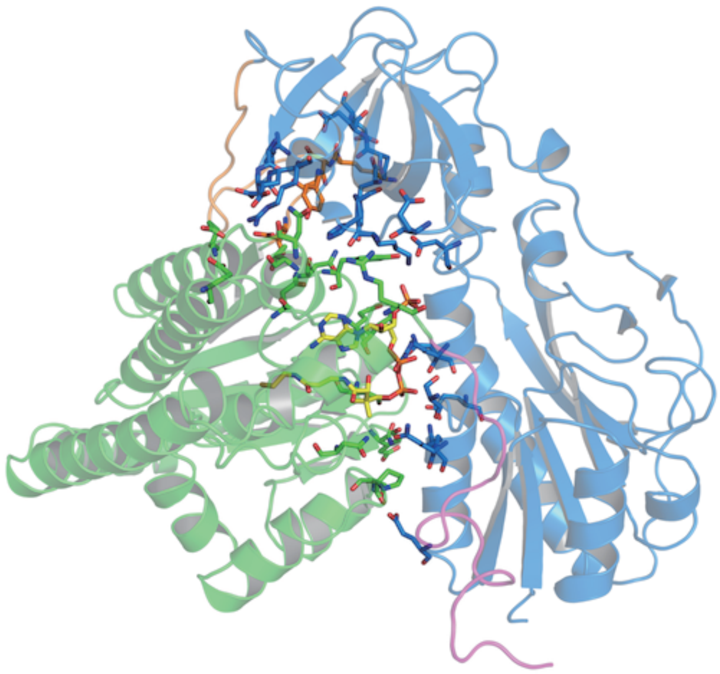

Supplementary Fig. 7. Hydrogen bonds in the interface between the N- and C-terminal domains. Amino acid residues and water molecules involved the hydrogen bonding interactions are shown in the stick model and red balls, respectively.

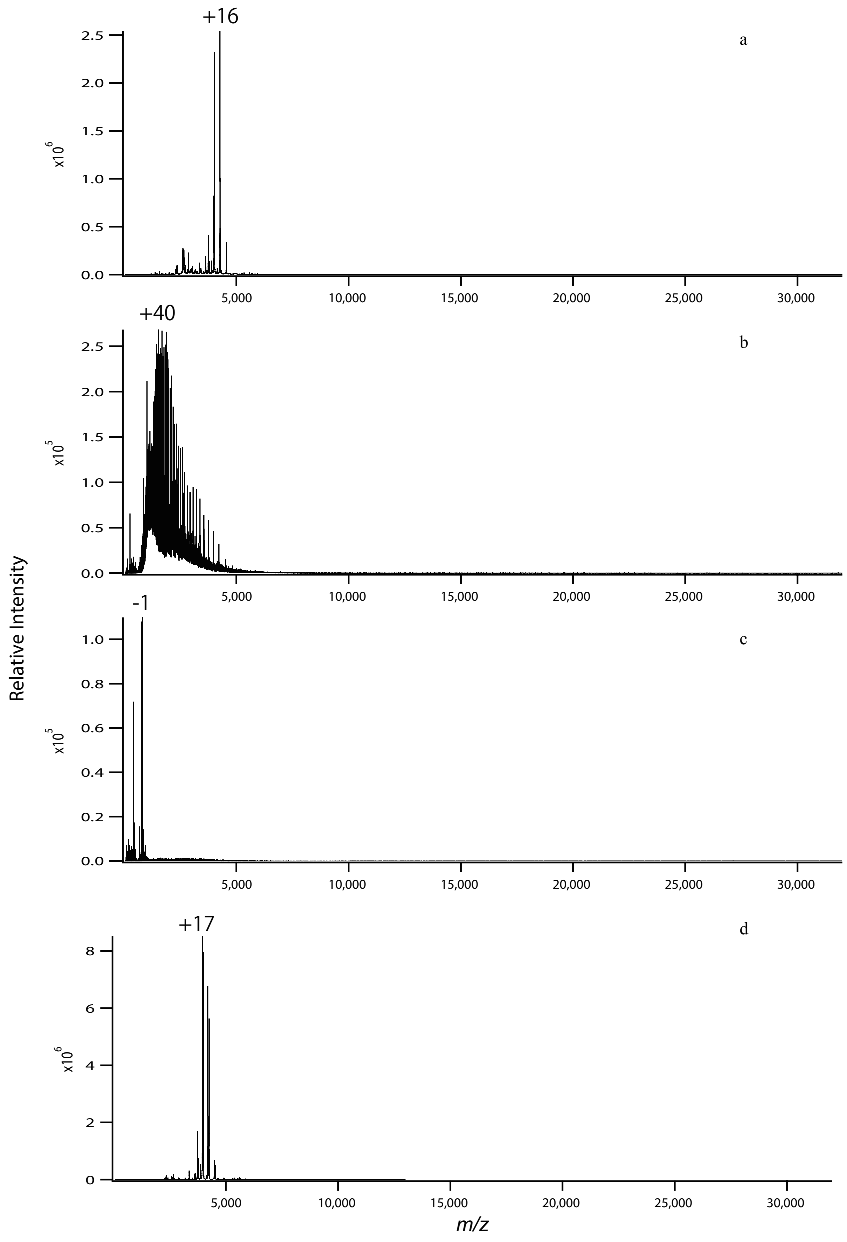

Supplementary Fig. 8. Full *m/z* range native mass spectra of Fig.6 in the main text.

a

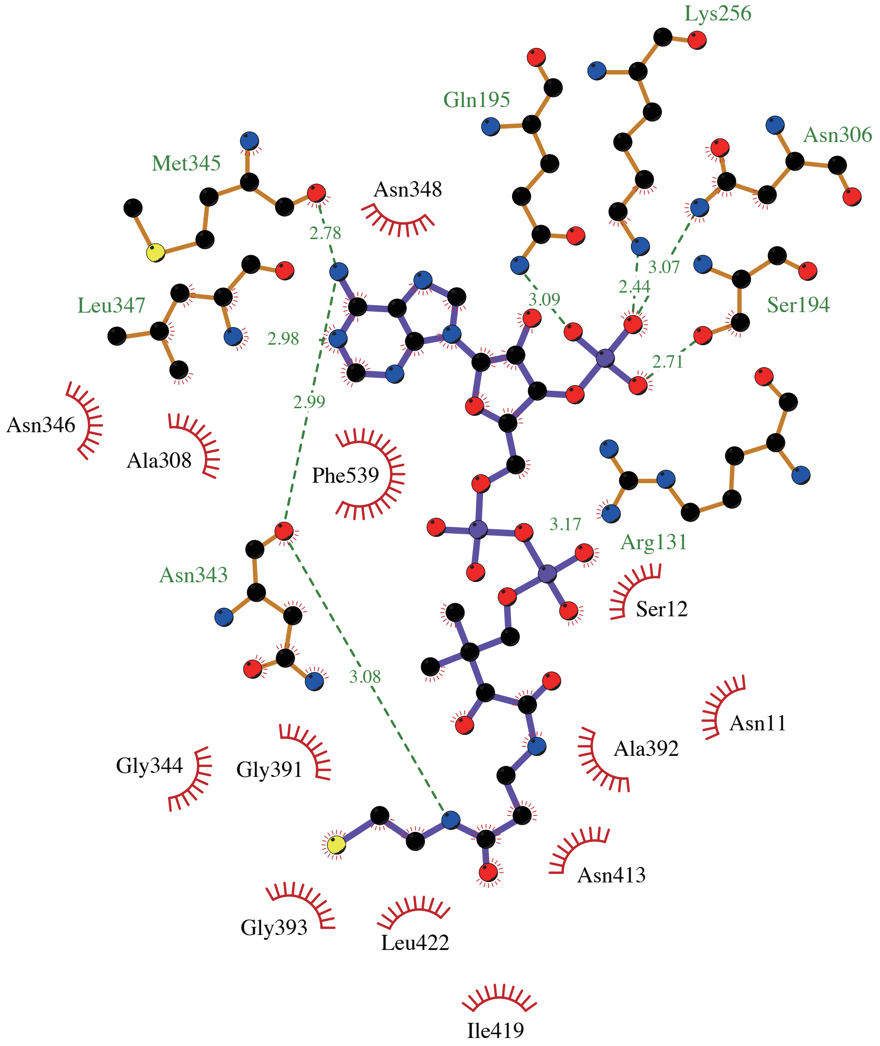
Lys256
Gln195
Asn306
Met345
Asn348
2.78
3.07
Leu347
3.09
2.44
Ser194
2.98
2.71
2.99
Asn346
Ala308
Phe539
3.17
Arg131
Asn343
Ser12
3.08
Asn11
Ala392
Gly344
Gly391
Asn413
Gly393
Leu422
Ile419

b

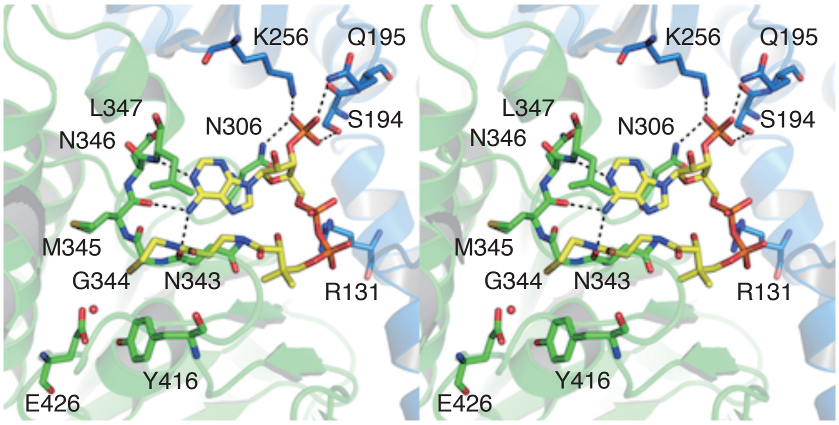
K256
Q195
L347
N306
S194
N346
M345
G344
N343
R131
Y416
E426
K256
Q195
L347
N306
S194
N346
M345
G344
N343
R131
Y416
E426

c

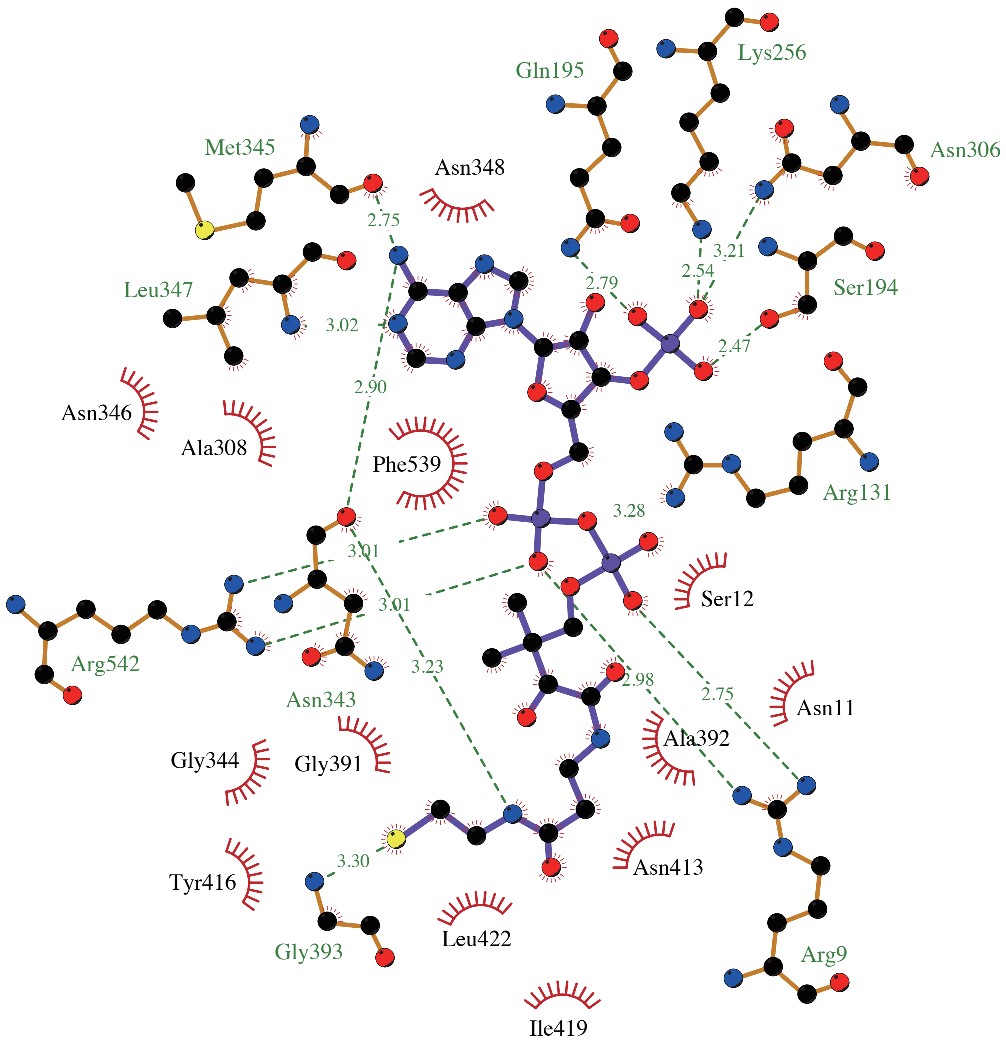

Supplementary Fig. 9. (a) Interaction diagrams (LigPlot+) for CoA and HypX interactions in the form I. (b) Stereo view of the CoA binding region in Form I. (c) LigPlot+ plot for CoA and HypX (form II).

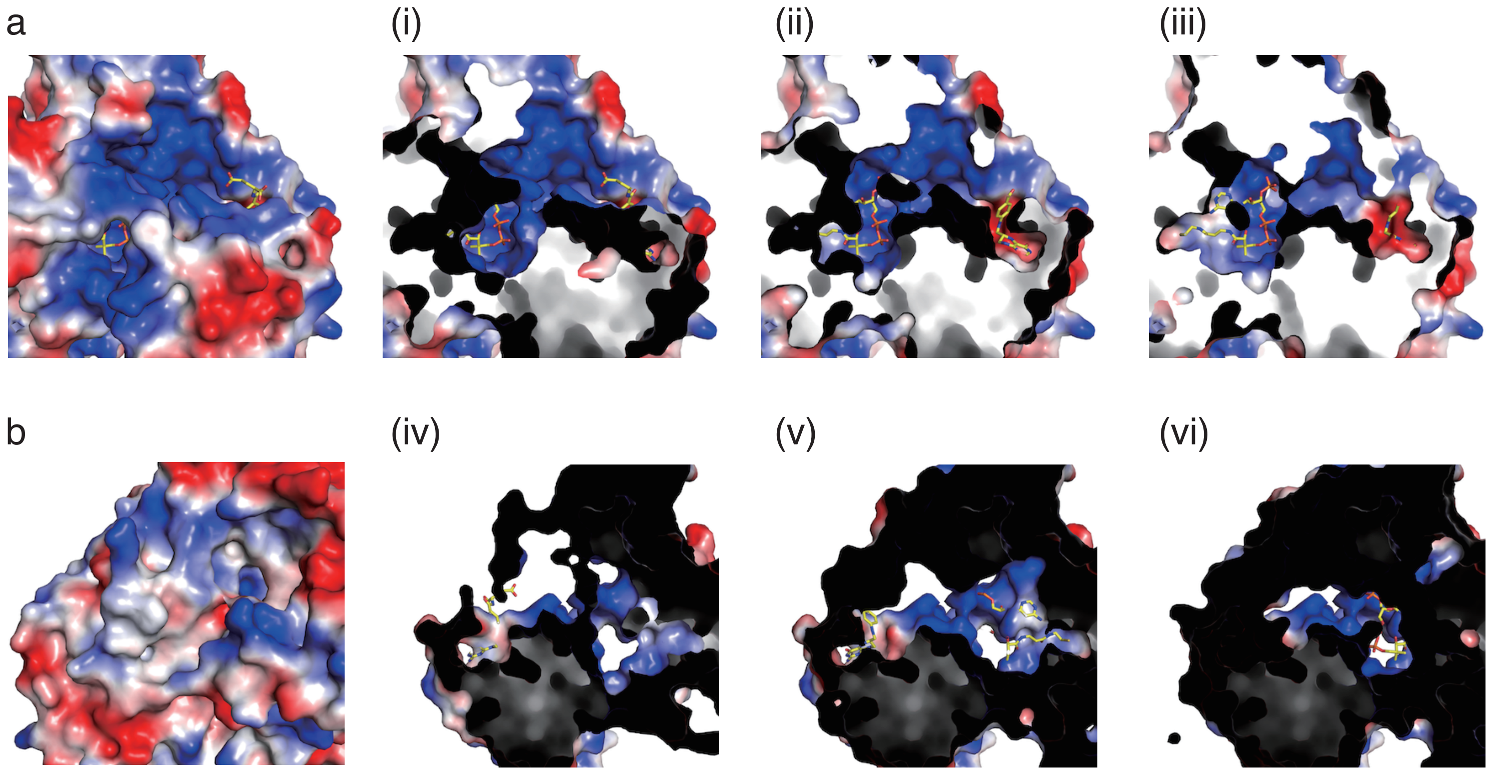

Supplementary Fig. 10. The cross-sectional views of HypX from the front (a) and the back (b). Positive and negative regions on the protein surfaces and the lining of the cavity are shown in blue and red, respectively. The orientation of (a) is the same as Fig. 5b. Cross sections from (i) to (iii) and from (iv) to (vi) are from the surface to the inside with arbitrary depths in (a) and (b), respectively.

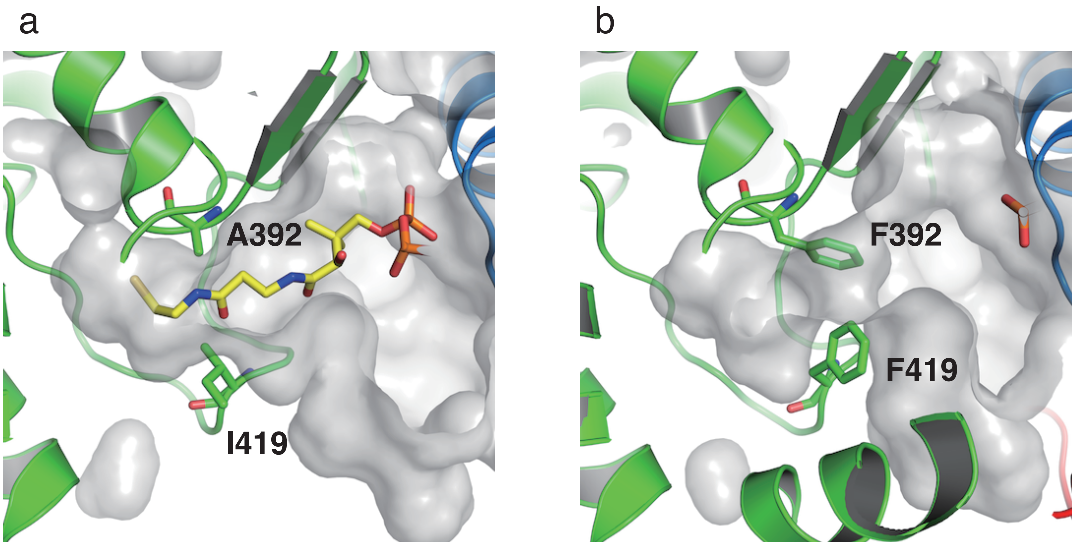

Supplementary Fig. 11. (a) Close-up view of the region around Ala392 and Ile419 in the cavity of wild type HypX. (b) Close-up view of the same region in the A392F-I419F variant. The cavity inside HypX is shown in gray.

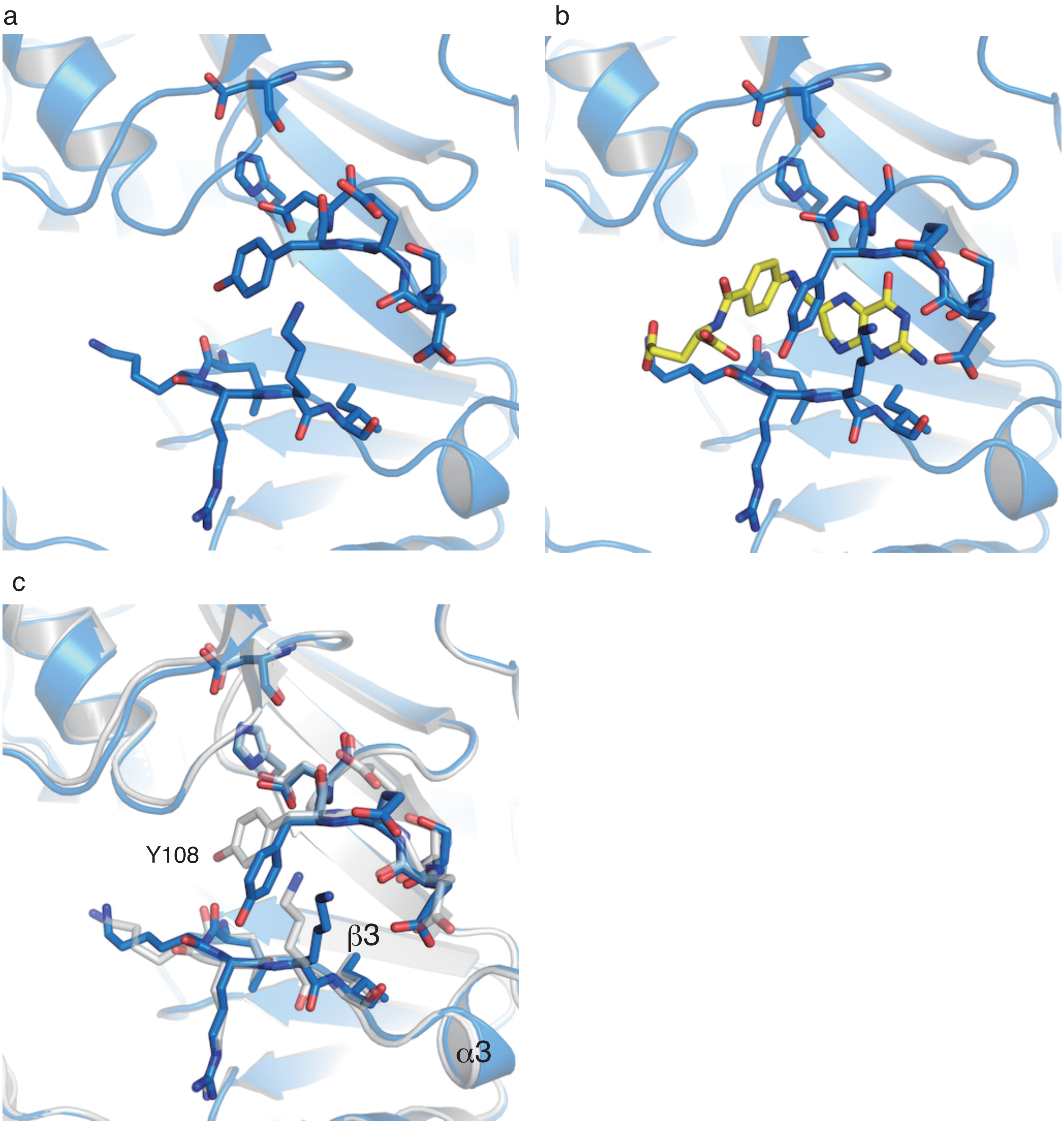

Supplementary Fig. 12. Conformational change of the A392F-I419F variant upon THF binding. Close-up view of the THF binding region (a) in the THF-free and (b) in the THF-bound forms. (c) Superposition of (a) and (b). THF is omitted for clarity. The structures of (a) and (b) are shown in blue and light gray, respectively.

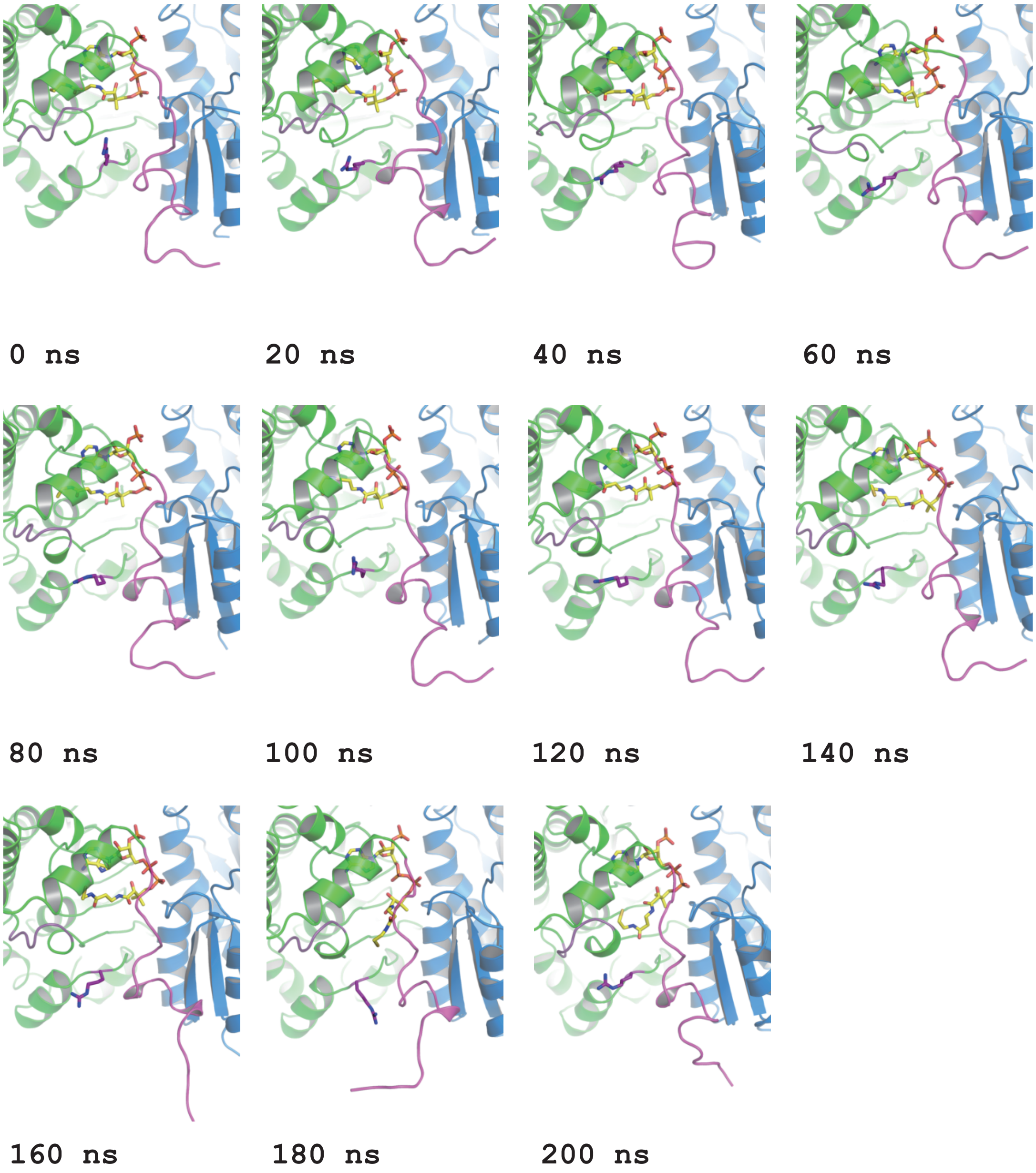

Supplementary Fig. 13. Snap shot views of the MD simulations per 20 ns. The β7 -β8 loop (residues 419-423) and Arg448 are shown in purple and the C-terminal tail is shown in magenta. CoA is shown in the stick model.

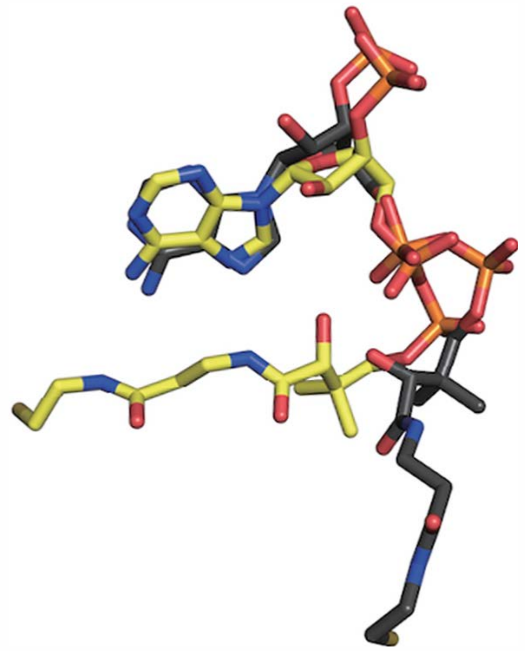

Supplementary Fig. 14. Superposition of the folded CoA (yellow and orange) in wild-type HypX and the partially folded CoA (black) observed in the MD simulation.

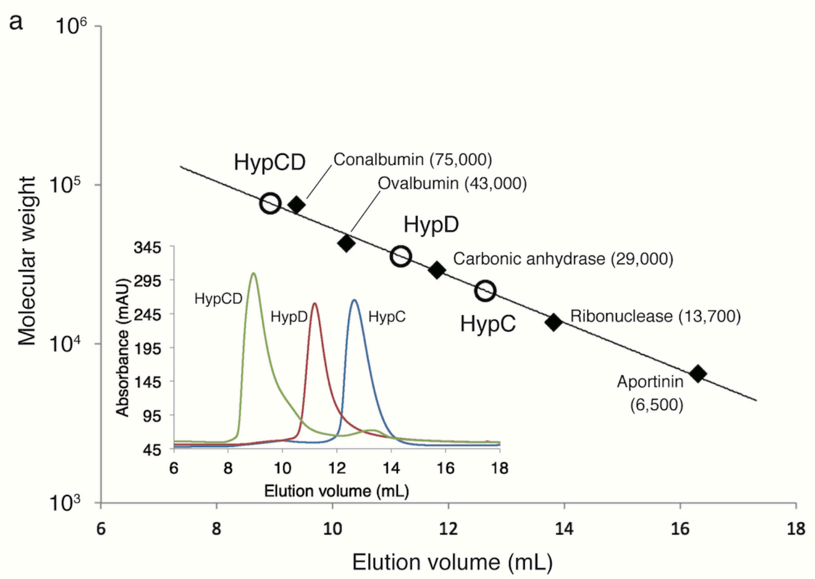

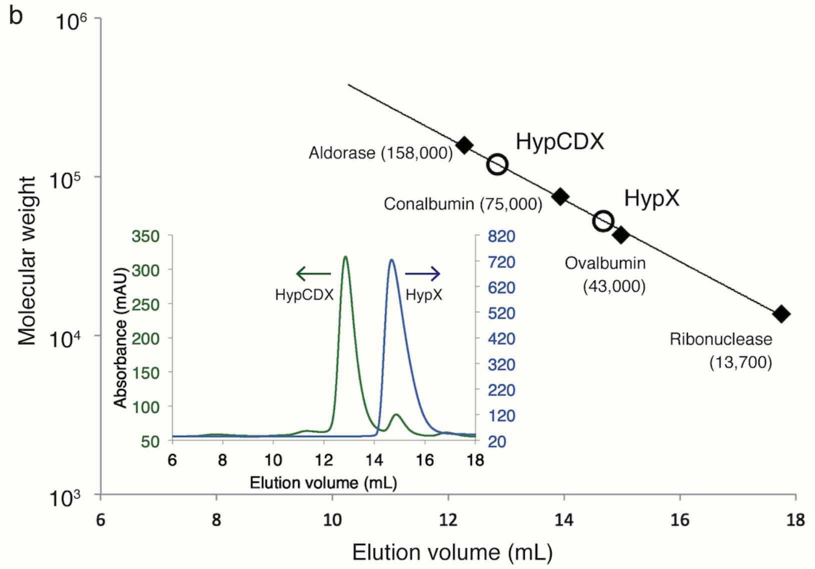

Supplementary Fig. 15. (a) Estimation of the mass of HypC, HypD, and HypC/HypD complex. The closed squares show the standard proteins. The vertical axis is in logarithmic scale. (Inset) Elution profiles of HypC, HypD, and HypC/HypD mixture from a Superdex75 10/300GL column. (b) Estimation of the mass of HypX and HypC/HypD/HypX complex. The vertical axis is in logarithmic scale. (Inset) Elution profiles of HypX and HypC/HypD/HypX mixture from a Superdex200 10/300GL column.

Supplementary Table 1. Hydrogen bonds in the interface between the N- and C-terminal domains. The amino acids in the direct hydrogen bond interactions (left) and the amino acids in the hydrogen bonds via water molecules (right).

N-terminal domain		C-terminal domain		Distance
Asn132	ND2	Asp339	OD1	2.84
Glu136	OE2	Arg407	NH1	2.93
Glu23	OE1	Ser452	OG	2.58
Glu23	OE2	Ser452	N	3.03
Gln15	OE1	Asn413	ND2	2.88
Tyr213	OH	Asn303	ND2	2.77
Asp193	OD1	Tyr305	OH	2.62
Lys256	NZ	Tyr305	OH	3.03
Lys256	O	Asn348	ND2	2.90
Gly253	O	Arg541	NH1	2.42
Arg261	NH2	Glu312	OE1	3.02

N-terminal domain		C-terminal domain	
Asn132	OD1	Glu338	O
Arg131	NH1	Asp339	O
Arg131	NH1	Asn389	ND2
Ser12	O	Asn389	ND2
Ser12	O	Asn389	ND2
Arg131	O	Arg407	NH2
Glu136	OE2	Arg407	NH2
Glu136	OE1	Glu408	O
Glu23	OE2	Pro450	O
Arg16	NH1	Gly409	O
Asn211	OD2	Asn303	O
His246	NE2	Asn346	ND2
Arg261	NH1	Asn352	ND2
Arg261	NH1	Thr349	O
Arg261	NH1	Glu359	OE2
Arg261	NH1	Glu312	OE1
Asp230	O	Glu288	OE2
Asp230	O	Arg316	NH2
Asp230	OD2	Glu288	OE1
Glu231	OE1	Arg316	NH1
Glu231	OE1	Ser310	OG

Supplementary Table 2. Primers used for mutagenesis, in which the mutated codon(s) is shown in red.

Mutation(s)	Primer(s)
R9A	5’- CGCATCCTGTTTCTGTCGTATGCATTCAATTCATTGTCAGCG
Q15A	5’- CGATTCAATTCATTGTCAGCGCGCTTGTATTGCGAAC<br>5’- GTTCGCAATACAAGCGCGCTGACAATGAATTGAATCG
R131A	5’- CGTAAAGCCTCAATTTATGCGAATGAAGTGACGGAAGGC
Q194A-Q195A	5’- GTCTATGCGTCTGACGCTGCACCAGGTGCCAGCAGCAAAG
N306A	5’- CATTCACTTCAACTTCTATGCTGGTGCGATGTCCACCGAGC
A392F	5’- CATGCAAGGCAATGCCGGTTTCGGCGGTGTTTTCCTCGC
I419F	5’- GAATCCCCACTACAAAAATTTCGGCAATCTGTATGGCTCC
R542A	5’- CGCTACTACTTTGTGCGCGCTAAACCCCACTTTCGTACTCC
