# Supplementary material for: Structural characterization of HypX responsible for CO biosynthesis in the maturation of NiFe-hydrogenase
Source: Commun Biol. 2019 Oct 18;2:385. doi: 10.1038/s42003-019-0631-z (PMC6802093; doi:10.1038/s42003-019-0631-z)
Supplement: Supplementary file 2 — Description of Additional Supplementary file [file 42003_2019_631_MOESM2_ESM.docx]

**Description of Additional Supplementary File**

File Name: Supplementary Movie 1

Description: The animation of one of the simulations for the conformational change of CoA in HypX.
